# Supplementary material for: Molecular hydrogen in minerals as a clue to interpret ∂D variations in the mantle
Source: Nat Commun. 2020 Jul 17;11:3604. doi: 10.1038/s41467-020-17442-8 (PMC7367874; doi:10.1038/s41467-020-17442-8)
Supplement: Supplementary file 1 — Supplementary Information [file 41467_2020_17442_MOESM1_ESM.pdf]

Supplementary information

Molecular Hydrogen in minerals as a clue to interpret ∂D variations in the mantle

B.N. Moine*[1], N. Bolfan-Casanova[2], I.B. Radu[1,3], D.A. Ionov[4], G. Costin[5], A.V. Korsakov[6], A.V. Golovin[6,7], O.B. Oleinikov[8], E. Deloule[9], JY. Cottin[1]

E-mail: **bertrand.moine@univ-st-etienne.fr (B. N. Moine)**

Supplementary Table 1: major elements composition of omphacite and garnet of eclogites and P-T estimations

	Obn108	Obn108	Obn108	Obn110	Obn110	Obn110	Obn111	Obn111	Obn111	Obn112	Obn112	Obn112	RV179	RV179	RV179	RV203	RV203	RV203
	Cpx	Cpx	Cpx	Cpx	Cpx	Cpx	Cpx	Cpx	Cpx	Cpx	Cpx	Cpx	Cpx	Cpx	Cpx	Cpx	Cpx	Cpx
SiO_2	52.17	50.33	52.57	52.19	49.06	50.06	51.90	52.08	50.13	51.18	50.48	50.12	50.01	50.80	50.66	55.35	55.42	55.51
TiO_2	0.31	0.20	0.33	0.05	0.11	0.10	0.07	0.04	0.03	0.04	0.05	0.12	0.01	0.02	0.00	0.19	0.07	0.17
Al_2O_3	10.61	14.42	9.90	14.84	19.37	17.62	14.84	14.13	16.93	15.26	16.02	16.01	19.81	19.77	19.75	5.66	5.71	5.66
Cr_2O_3	0.11	0.17	0.10	0.08	0.07	0.04	0.03	0.02	0.05	0.05	0.05	0.04	0.13	0.14	0.14	0.08	0.08	0.07
MgO	11.63	10.06	11.99	9.53	8.35	8.97	9.57	9.98	8.98	9.47	9.25	9.30	6.48	6.42	6.39	6.70	6.62	6.63
FeO	2.01	1.80	2.09	0.94	1.04	0.96	0.91	0.86	0.87	1.21	1.18	1.22	0.83	0.76	0.73	0.07	0.12	0.10
MnO	0.04	0.04	0.01	0.00	0.01	0.00	0.07	0.00	0.02	0.01	0.00	0.00	0.02	0.00	0.04	12.05	11.88	11.69
CaO	19.53	18.61	19.41	17.81	18.54	19.09	18.11	18.44	18.30	18.82	18.93	18.99	15.03	14.86	14.68	16.32	16.29	16.08
Na_2O	3.53	3.91	3.44	4.34	3.99	3.85	4.40	4.31	3.96	4.07	3.94	3.69	6.61	6.73	6.62	3.92	3.90	3.91
K_2O	0.01	0.00	0.06	0.01	0.00	0.07	0.00	0.01	0.01	0.00	0.01	0.00	0.02	0.03	0.03	0.00	0.05	0.03
Total	99.95	99.55	99.90	99.79	100.56	100.77	99.90	99.86	99.27	100.11	99.91	99.48	99.06	99.62	99.10	100.35	100.13	99.85
XCa	0.52	0.55	0.51	0.56	0.60	0.59	0.56	0.56	0.58	0.57	0.58	0.58	0.61	0.61	0.61	0.64	0.64	0.63
XFe	0.04	0.04	0.04	0.02	0.03	0.02	0.02	0.02	0.02	0.03	0.03	0.03	0.03	0.02	0.02	0.00	0.00	0.00
XMg	0.43	0.41	0.44	0.42	0.37	0.39	0.41	0.42	0.40	0.40	0.39	0.39	0.36	0.37	0.37	0.36	0.36	0.36
T (°C) Krogh-Ravna (2000)		1137			954			923			982			986			861	
P (GPa)		4.1			3.2			3.1			3.3			2.7			2.2	

	RV233	RV233	RV233	RV360	RV360	RV360	RV377	RV377	RV377	RV469	RV469	RV469	RV488	RV488	RV488	RV513	RV513	RV513
	Cpx	Cpx	Cpx	Cpx	Cpx	Cpx	Cpx	Cpx	Cpx	Cpx	Cpx	Cpx	Cpx	Cpx	Cpx	Cpx	Cpx	Cpx
SiO_2	55.04	54.72	54.85	55.54	55.54	55.68	51.11	51.26	51.21	55.62	55.72	55.75	55.64	55.41	55.25	56.06	55.59	55.60
TiO_2	0.21	0.16	0.22	0.18	0.21	0.17	0.13	0.08	0.08	0.17	0.16	0.22	0.24	0.24	0.28	0.31	0.32	0.34
Al_2O_3	8.56	8.79	8.91	2.00	2.17	2.27	10.60	10.24	10.48	10.34	10.01	9.69	2.90	2.91	2.78	3.72	3.92	3.78
Cr_2O_3	0.06	0.09	0.08	0.39	0.37	0.33	0.13	0.13	0.16	0.05	0.04	0.08	0.44	0.41	0.46	0.16	0.25	0.21
MgO	4.74	4.84	5.04	3.06	2.28	2.75	2.62	2.67	2.66	8.59	8.60	8.62	2.70	3.21	2.91	3.30	3.44	3.63
FeO	0.00	0.01	0.00	0.09	0.06	0.09	0.01	0.05	0.05	6.09	5.91	5.79	0.06	0.11	0.08	0.07	0.10	0.11
MnO	9.93	9.79	9.79	16.81	17.31	16.90	12.20	12.25	12.22	0.02	0.09	0.00	16.86	16.99	17.15	15.61	15.35	15.31
CaO	16.28	16.12	16.01	20.18	20.44	20.68	20.65	20.81	20.95	13.78	13.69	13.87	18.98	18.54	18.73	18.67	18.43	18.02
Na_2O	4.80	4.76	4.87	1.43	1.40	1.41	2.08	2.13	2.07	5.96	5.81	5.64	1.93	1.97	2.03	2.55	2.68	2.62
K_2O	0.00	0.02	0.00	0.02	0.01	0.03	0.00	0.02	0.00	0.00	0.00	0.00	0.06	0.07	0.07	0.05	0.01	0.02
Total	99.62	99.31	99.77	99.69	99.79	100.31	99.53	99.64	99.88	100.63	100.04	99.66	99.79	99.85	99.74	100.51	100.10	99.63
XCa	0.71	0.71	0.70	0.82	0.86	0.84	0.85	0.85	0.85	0.45	0.45	0.46	0.83	0.80	0.82	0.80	0.79	0.78
XFe	0.00	0.00	0.00	0.00	0.00	0.00	0.00	0.00	0.00	0.16	0.15	0.15	0.00	0.00	0.00	0.00	0.00	0.00
XMg	0.29	0.29	0.30	0.17	0.13	0.16	0.15	0.15	0.15	0.39	0.40	0.39	0.16	0.19	0.18	0.20	0.21	0.22
T (°C) Krogh-Ravna (2000)		987			805			1104			1109			946			991	
P (GPa)		2.7			2.1			3.4			3.1			2.6			2.8	

Supplementary Table 2: Water content and hydrogen isotope composition of heated omphacites based on TC/EA-IRMS, FTIR analyses.

sample	n	∂D ‰ vSMOW	±‰	H_2O (ppm) TC/EA-MS	1 SD	n	H_2O_{tot} (ppm) FTIR 3000-3800 cm^{-1}	±30%	Abs norm 5200cm^{-1}	H2O_{mol} (ppm) FTIR 5200cm^{-1}	±30%	Abs Int norm 4000-4300cm^{-1}	1 SD	H_2 (ppm)	1SD	1/H2
Obn108_400	4	-106	3	2778	132	9	1023	307	0.0822	239	71.7	10.0	8.6	195	37	0.00513
Obn110_400	4	-120	2	4122	149	9	633	190	0.0650	216	64.8	1.5	0.5	388	27	0.00258
Obn111_400	4	-117	2	4880	410	9	975	293	0.0864	286	85.8	19.0	8.4	434	56	0.00230
Obn112_250	4	-120	2	4895	120											
Obn112_400	4	-116	4	4446	200	9	802	241				19.1	3.4	405	35	0.00247
Obn112_500	4	-112	2	4190	260	9	626	188	0.0476	158	47.4	6.5	5.6	396	36	0.00253
Obn112_600	4	-107	2	3814	27	9	565	170	0.0508	169	50.7	5.9	4.5	361	19	0.00277
Kovacs et al 2016																
Nushan	cpx4	-45	2	1152	39		302	90.6						94	32.9	0.01059
Nushan	cpx6	-62	7	1457	81		180	54						142	32.4	0.00705
Nushan	cpx11	-58	4	1206	51		99	29.7						123	19.7	0.00813
Nushan	cpx13	-86	4	1882	322		20	6						207	35.8	0.00483

Supplementary Figure 1

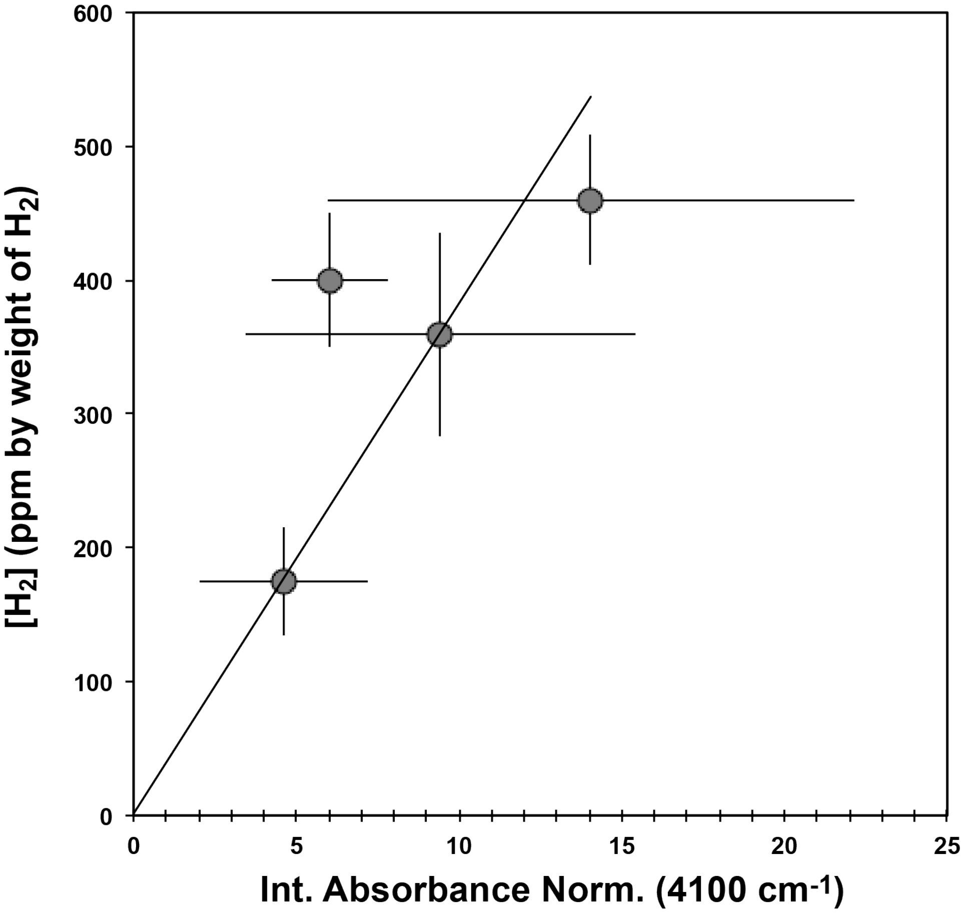

Supplementary Figure 1: Calculated molecular H_2 versus Integrated Absorbance normalized to 1 cm corresponding to the 4100 cm^{-1} peak. With the exception of one sample (Obn110), all samples align very well on a straight line passing through zero. This demonstrates that the 4100 cm^{-1} band is indeed due to H_2 and not an overtone of the silicate lattice vibration. It also shows that little molecular water participates to the bulk H_2O measurement by TC/EA-IRMS, otherwise it would yield random values of the absorbance coefficient.

Supplementary Figure 2

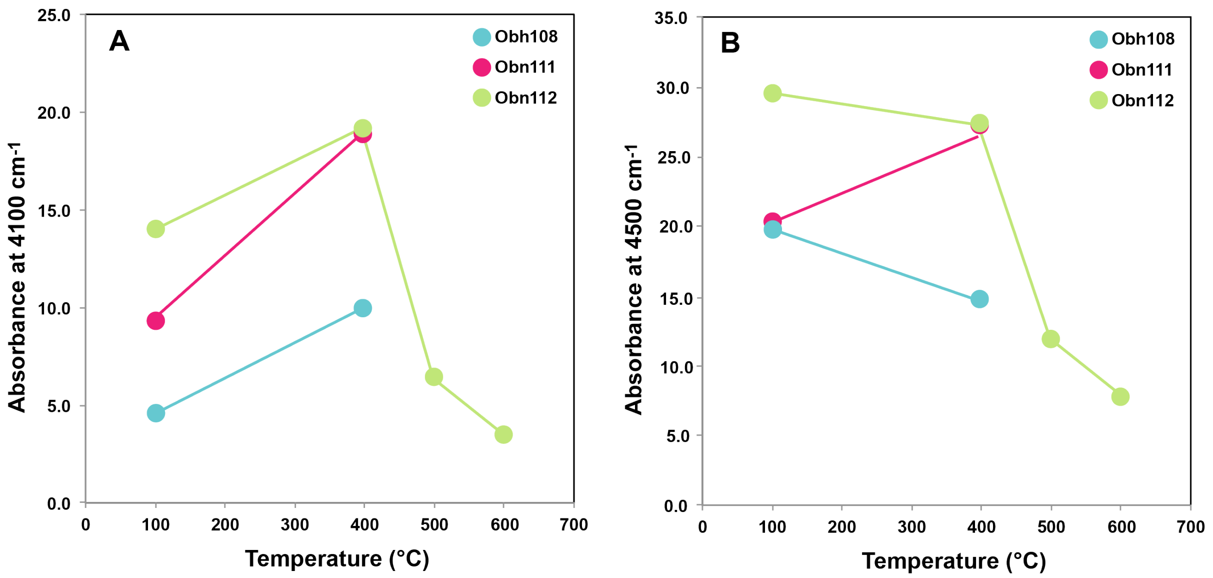

Supplementary Figure 2: Integrated normalized absorbance at (A) 4100 cm^{-1} and (B) 4500 cm^{-1} versus the temperature at which the samples were heated.

Supplementary Figure 3

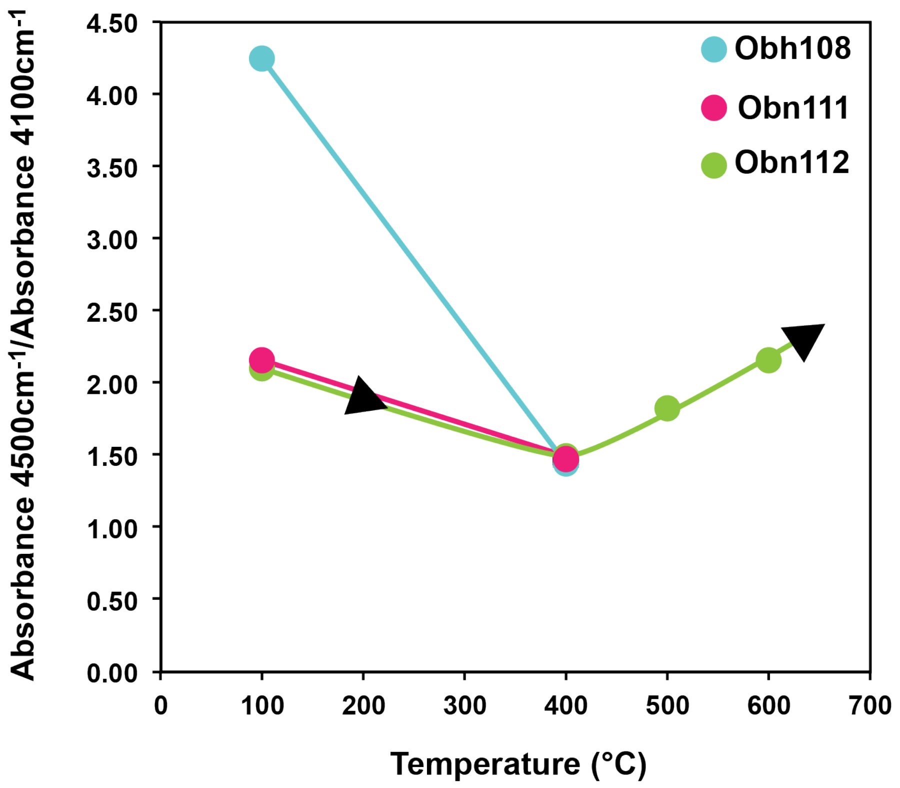

Supplementary Figure 3: Ratio of integrated normalized absorbances at 4500 cm^{-1} and 4100 cm^{-1} versus the temperature at which the samples were heated.

Supplementary Figure 4

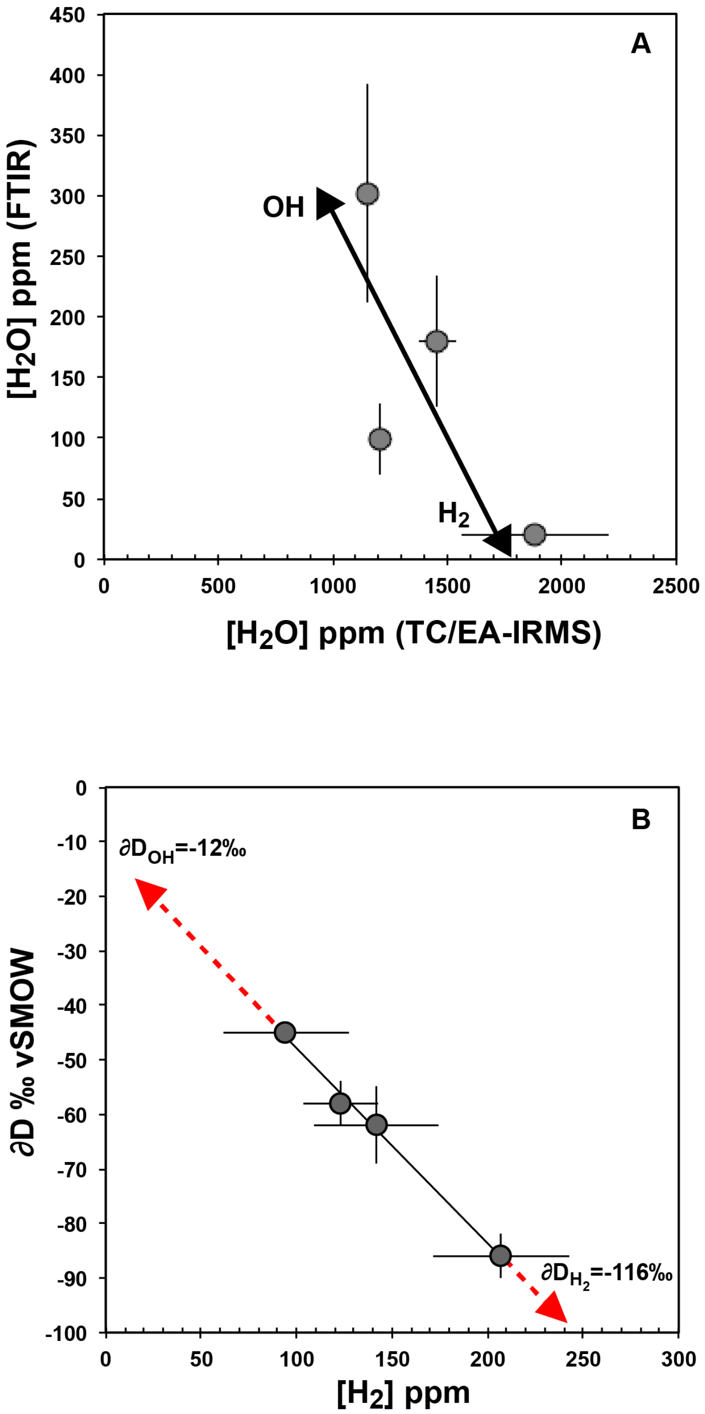

Supplementary Figure 4: (A) water and (B) molecular H_2 contents (ppm) versus ∂ D (‰ V-SMOW) from Nushan augite in [26]
